# Hijacking Host Communication: The Central Role of Extracellular Vesicles in Infectious Disease Pathogenesis

**DOI:** 10.3390/cells15181708

**Published:** 2026-09-20

**Authors:** Patrick Santos, Fausto Almeida

**Affiliations:** 1 Department of Internal Medicine, Ribeirão Preto Medical School, University of São Paulo, Ribeirão Preto 14048-900, SP, Brazil; patricksantos@usp.br; 2 Department of Biochemistry and Immunology, Ribeirão Preto Medical School, University of São Paulo, Ribeirão Preto 14048-900, SP, Brazil

**Keywords:** intercellular communication, chronic infection, immune evasion, virulence factors, biomarkers

## Abstract

**Highlights:**

**What are the main findings?**

- Extracellular vesicles constitute a conserved communication network that redefines host–pathogen interactions across infectious diseases.
- EV cargo integrates immune modulation, pathogen persistence, and tissue pathology through shared mechanisms across distinct pathogen kingdoms.

**What are the implications of the main findings?**

- This review provides a unified cellular framework connecting EV biology from protozoan, viral, and bacterial infections.
- Targeting EV-mediated communication opens new opportunities for precision diagnostics, vaccines, and host-directed therapies.

**Abstract:**

Extracellular vesicles (EVs) have emerged as fundamental mediators of intercellular communication during infectious diseases, acting as vehicles for proteins, lipids, and nucleic acids that profoundly influence host–pathogen interactions. In recent years, accumulating evidence has revealed that pathogens from distinct biological kingdoms, including protozoan parasites, viruses, and bacteria, either release their own EVs or exploit host-derived EVs to manipulate immune responses, promote infection, and sustain chronic disease. This review provides a comprehensive and integrative overview of the roles of EVs in infectious diseases, with particular emphasis on immune modulation, immune evasion, pathogenesis, and translational applications. We summarize current knowledge on EV-mediated communication in infections caused by major protozoan parasites (*Trypanosoma cruzi*, *Plasmodium* spp., *Toxoplasma gondii*, and *Leishmania* spp.), highlighting how parasite- and host-derived EVs regulate inflammatory responses, facilitate intracellular survival, and shape disease outcomes. We further examine viral infections, including HIV, HPV, HCV, and airborne respiratory viruses such as SARS-CoV-2, where EVs contribute to viral persistence, immune dysfunction, oncogenesis, and long-term sequelae. In addition, we discuss bacterial infections, focusing on *Mycobacterium tuberculosis*, *Streptococcus pneumoniae*, and *Helicobacter pylori*, illustrating how EVs can function both as vectors of virulence factors and as components of host defense mechanisms. Finally, we explore the emerging translational potential of EVs as non-invasive biomarkers for disease diagnosis and prognosis, as well as their application as therapeutic and vaccine platforms. By integrating findings across diverse pathogens, this review highlights extracellular vesicles as widely implicated and dynamic contributors of infectious disease biology and underscores their relevance as targets and tools for next-generation diagnostic and therapeutic strategies.

## 1. Introduction

Diseases caused by pathogenic microorganisms continue to pose a substantial global health burden, largely because pathogens establish complex and dynamic interactions with host cells that drive immune activation, immune evasion, tissue damage, and long-term clinical sequelae. Increasing evidence indicates that extracellular vesicles (EVs) are integral components of this host–pathogen dialogue, functioning as structured and regulated platforms for the transfer of bioactive molecules rather than passive byproducts of infection.

The recognition of EVs as genuine communication vehicles, rather than incidental cellular debris, has followed a comparatively long and often overlooked historical trajectory. Vesicular structures were first inferred in 1946, when a procoagulant particulate fraction, later termed “platelet dust,” was isolated from plasma by high-speed centrifugation [1]. Decades later, vesicles released during reticulocyte maturation were characterized and named “exosomes,” initially interpreted as a mechanism for discarding obsolete membrane proteins during erythrocyte differentiation [2]. The conceptual shift toward EVs as active mediators of intercellular signaling was catalyzed in 1996, when B-lymphocyte-derived exosomes were shown to carry MHC class II molecules and to elicit antigen-specific T-cell responses [3]. Only in the past two decades, with the maturation of high-resolution proteomic, transcriptomic, and imaging technologies, has this communication paradigm been extended to host–pathogen biology, revealing that protozoa, viruses, and bacteria not only release their own EVs but also actively remodel host EV biogenesis to their advantage.

At the molecular level, EV-mediated communication in infectious diseases converges on a limited set of conserved biogenesis and uptake pathways, regardless of the pathogen involved. Intraluminal vesicles destined to become exosomes are generated within multivesicular bodies through ESCRT-dependent and ESCRT-independent, ceramide-driven budding, whereas microvesicles bud directly from the plasma membrane; both vesicle classes converge functionally once released [4]. Recipient cells internalize EVs mainly through clathrin- or caveolin-mediated endocytosis, macropinocytosis, or direct membrane fusion, delivering cargo, including proteins, lipids, microRNAs, and, for several pathogens, nucleic acids or virulence factors, into the cytoplasm of target cells. Pathogens exploit this machinery in two broad, non-mutually exclusive ways: by releasing their own EVs carrying immunomodulatory or virulence cargo, or by hijacking host EV biogenesis so that infected cells secrete vesicles enriched in pathogen-derived molecules. As detailed in the sections below, this shared molecular logic underlies phenomena as diverse as cytokine induction by protozoan EVs, viral protein packaging into host exosomes, and bacterial outer membrane vesicle-mediated immune evasion, even though the specific cargo and downstream consequences differ markedly across pathogen kingdoms. Different EV subtypes may show overlapping functional properties once released into the extracellular space, but their composition, targeting, kinetics, and biological activity can also differ substantially; functional equivalence should therefore not be assumed.

Several reviews have addressed EV biology within individual pathogen groups, typically focusing exclusively on protozoan parasites, on a specific viral family, or on bacterial pathogens considered in isolation. What remains comparatively underexplored is a systematic, side-by-side comparison of how EV-mediated strategies converge and diverge across the three major pathogen kingdoms. In this review, we selected four protozoan parasites (*Trypanosoma cruzi*, *Plasmodium* spp., *Toxoplasma gondii*, and *Leishmania* spp.), four viruses (HIV, HPV, HCV, and SARS-CoV-2), and three bacteria (*Mycobacterium tuberculosis*, *Streptococcus pneumoniae*, and *Helicobacter pylori*) because each ranks among the pathogens with the largest global health burden within its group and, critically, each has generated a sufficiently mature body of EV-specific literature to support mechanistic comparison rather than superficial cataloguing. Beyond summarizing pathogen-specific findings, we explicitly compare the mechanisms identified across these three kingdoms in a dedicated synthesis section (Section 5), highlighting recurring themes, such as the convergent use of EVs for immune evasion and the cargo-dependent duality of outcome, as well as kingdom-specific divergences that remain unresolved.

Across a wide range of infectious diseases, EVs have emerged as conserved mediators through which pathogens and infected host cells modulate immune responses and reshape tissue microenvironments. By transporting proteins, lipids, and nucleic acids, EVs can amplify inflammation, suppress antimicrobial immunity, facilitate pathogen persistence, or, in some contexts, contribute to host defense. This duality highlights EVs as dynamic regulators whose biological impact is dictated by cargo composition, cellular origin, and infection stage.

For clarity, this review distinguishes three EV populations where relevant: pathogen EVs (vesicles released directly by the pathogen itself, e.g., parasite exosome-like vesicles or bacterial outer-membrane vesicles), infected-host-cell EVs (EVs released by host cells in response to infection, which may carry pathogen-derived cargo but originate from host membranes), and uninfected-host-cell EVs (EVs from bystander or non-infected host cells, used here as comparators). Unless otherwise specified, associations between EV cargo (e.g., specific miRNAs or proteins) and a recipient-cell phenotype reported in this review reflect correlative evidence from expression or co-culture studies; we note explicitly where loss-of-function approaches (cargo depletion, inhibitor treatment, rescue experiments, pathogen mutants, or receptor blockade) have established a functional or causal role.

In this review, we synthesize current knowledge on EV-mediated communication in infectious diseases, structured around three major pathogen groups: protozoa, viruses, and bacteria. By integrating mechanistic insights with emerging translational perspectives, we highlight extracellular vesicles as unifying elements of infectious disease biology and discuss their potential to inform the development of biomarkers, vaccines, and host-directed therapies.

### Literature Search and Pathogen Selection Strategy

Relevant literature was identified through systematic searches of PubMed/MEDLINE, Scopus, and Web of Science, using combinations of the terms “extracellular vesicles,” “exosomes,” “microvesicles,” “host–pathogen interaction,” and the name of each pathogen or disease discussed. Searches were restricted to peer-reviewed articles published in English, with the final search conducted in June 2026. Original research articles, systematic reviews, and clinical trial reports were considered; conference abstracts, preprints, and non-peer-reviewed sources were excluded unless no peer-reviewed alternative was available. Pathogens were selected based on (i) global disease burden as reported by WHO/CDC surveillance data, (ii) the existence of a critical mass of peer-reviewed EV-specific literature (≥10 primary studies), and (iii) representation of protozoan, viral, and bacterial infection classes, so that the comparative framework in Section 5 would not be biased toward a single pathogen kingdom. We acknowledge that this strategy, while systematic, does not constitute a formal systematic review (e.g., PRISMA), and we have added a statement to this effect together with a note on the corresponding limitation in the Conclusions.

## 2. Protozoa: Extracellular Vesicles as Modulators of Host Immunity and Parasite Persistence

**Figure 1:**
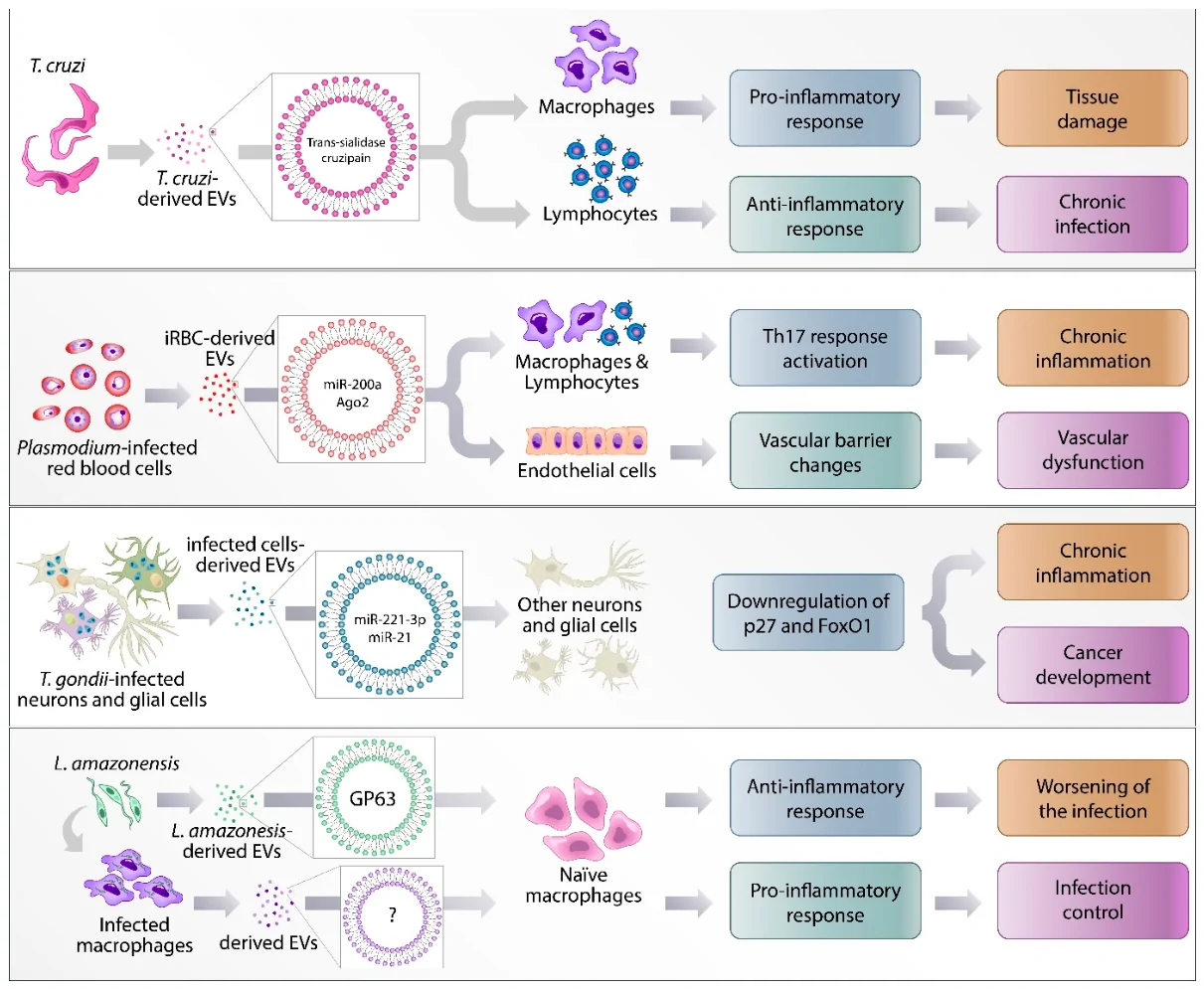
Extracellular vesicle-mediated immune modulation by protozoan parasites. Schematic representation of how extracellular vesicles (EVs) derived from protozoan parasites or parasite-infected host cells modulate host immune responses and disease outcomes. *Trypanosoma cruzi* releases EVs enriched with virulence factors such as trans-sialidase and cruzipain, which differentially interact with macrophages and lymphocytes, promoting early pro-inflammatory responses associated with tissue damage, followed by anti-inflammatory signaling that facilitates immune evasion and chronic infection. In malaria, EVs derived from *Plasmodium*-infected red blood cells (iRBCs) carry immunomodulatory miRNAs, including miR-200a and Ago2, targeting macrophages, lymphocytes, and endothelial cells, leading to Th17 activation, chronic inflammation, vascular barrier disruption, and vascular dysfunction. During *Toxoplasma gondii* infection, EVs released from infected neurons and glial cells contain miRNAs such as miR-221-3p and miR-21, which are transferred to neighboring neural cells, downregulate tumor suppressors p27 and FoxO1, sustain chronic inflammation, and potentially contribute to cancer development. In *Leishmania amazonensis* infection, parasite-derived EVs enriched in GP63 and EVs released from infected macrophages exert divergent effects on naïve macrophages, inducing either anti-inflammatory responses that worsen infection or pro-inflammatory responses that promote parasite control. Collectively, this figure highlights the context-dependent and dual roles of protozoan-derived and infection-induced EVs in shaping immune responses, tissue pathology, and infection outcomes.

Protozoan parasites represent a worldwide threat to human health, due to their ability to modulate the immune system and to manipulate host cell functions, sustaining the infection and continuing their life cycle in the human body [5]. Although there are limited entries in the literature, EVs released by protozoans have been documented to play a role in pathogenesis. Several studies indicate that EVs are involved in immune evasion and the modulation of host cell responses. There are four protozoans that have more information about their EVs’ intercellular communication during the infection. *Trypanosoma cruzi* is an intracellular parasite known for causing a variety of infectious diseases, most notably Chagas disease. There are different species of *Plasmodium*, which are the parasite linked to malaria, a tropical endemic disease. *Toxoplasma gondii* is responsible for toxoplasmosis, a non-contagious infection that can disrupt the host’s immune system. Finally, *Leishmania* spp. is a parasite genus responsible for causing a group of diseases, known as leishmaniasis, which often manifest as cutaneous lesions (Figure 1).

### 2.1. Trypanosoma cruzi

*Trypanosoma cruzi* (*T. cruzi*) is the etiological agent of Chagas disease, a prevalent endemic health burden in South and Central Americas [6]. However, there is an urgent rise in cases in North America, especially in the United States of America [7]. The acute stage of Chagas disease is often asymptomatic, showing few signs such as fever and chagoma, an inflamed swelling area created by the triatomine bug’s bite. If not treated, this infection can transition to the chronic stage, which is also asymptomatic on normal physical examination; however, it can cause cardiac complications detected by electrocardiogram [8]. According to a recent meta-analysis, prevalence of different forms of cardiomyopathy was observed in more than 40% of infected individuals [9]. At the cellular level, chronic *T. cruzi* infection generates potent innate and adaptative immune responses that can trigger tissue damage and the development of an inflammatory state in the host, due to the bystander effect and molecular mimicry [10].

Recently, more studies about EVs shed by *T. cruzi* support the role of these vesicles during the parasite’s communication with host immune cells (Figure 1). Cronemberg-Andrade et al. demonstrated that EVs from *T. cruzi* interacting with THP-1 cells activated Toll-like receptor-2 (TLR2) signaling and elevated TNF-α, IL-6, and IL-1β expression [11]. These macrophages were also found carrying and transporting parasite proteins in their EVs, which indicates a potential mechanism that amplifies inflammation and potential tissue damage. However, this initial host proinflammatory state triggered by *T. cruzi* and its EVs is attenuated as the infection persists, enabling the parasite to evade immune surveillance in the chronic stages of Chagas disease [12,13,14]. This process serves as an effective mechanism for enhancing host-cell invasion and sustaining infection. For example, Nogueira et al. reported that EVs released by *T. cruzi* trigger a differential expression pattern in immune cells during the innate and adaptive phases in infected mice, while macrophages promoted infection via TLR2 signaling after being exposed to trypomastigotes EVs, and lymphocytes CD4^+^ and CD8^+^ displayed an anti-inflammatory pattern, by increasing the expression of IL-10 [15].

In addition, circulating EVs from patients with different stages of Chagas disease incubated with human macrophages reduced IFN-γ expression in correlation with the increasing severity of cardiac complications [16]. These results can be correlated to Moreira et al., in which they demonstrated with several analyses, including liquid chromatography/mass spectrometry, the *T. cruzi* phenomenon of remodeling EVs’ composition at different stages of infection [17]. Altogether, *T. cruzi* employs multiple strategies to evade the host immune system, with the release of EVs that modulate immune responses being one of them. This host-response modulation by EVs is different for each life stage of the parasite, a topic effectively reviewed by Torrecilhas et al. [18]. However, there is a lack of information concerning *T. cruzi*-derived EV cargo; small RNA profiling revealed that the aligned reads could not be assigned to any known gene [19]. A proteomic analysis showed that *T. cruzi*-derived EVs are enriched with virulence factors, such as trans-sialidase (TS) and cruzipain [16]. The TS effect on the immune system is correlated with reducing T CD8^+^ cell activation and inducing T cell apoptosis in general, which leads to high levels of parasitemia and mortality in infected individuals [20]. Over time, the parasite becomes increasingly difficult for the host’s immune defenses to detect, and EVs play an essential role not only in the initial innate immune response, but also in the subsequent immune evasion processes that facilitate the growth of *T. cruzi* within the host.

### 2.2. Plasmodium spp.

Malaria is an endemic parasitic disease in subtropical and tropical regions around the world, leaving nearly 40% of global population at risk of *Plasmodium* spp. infection [21]. Malaria pathogenesis is a complex process that involves many different settings and outcomes from the interaction between humans and mosquitoes infected with *Plasmodium* spp. throughout its evolution [22]. According to Milner Jr., in most cases, every person infected with *Plasmodium* goes through the complete parasite life cycle without knowing. However, disease signs, such as fever, chills, headache, result from an imbalance in the interactions between the parasite and the host [23]. Following the infection, the host immune system triggers efficient responses to address the parasite; however, *Plasmodium* sporozoites counteract these defenses by undergoing a complex liver and blood stage cycle. At this point, the parasite, in the merozoite form, interacts with host cells to disrupt cellular machinery and to evade immunological mechanisms, facilitating progression of the infection to various tissues [24].

As expected, *Plasmodium* species utilize EVs to facilitate communication with host cells during infection (Figure 1). Although the isolation, characterization and profiling of EVs derived from *Plasmodium* species and malaria-infected cells have been investigated over the last two decades, their specific function in the disease infection has yet to be clearly elucidated. Some of these studies revealed that EVs derived from infected red blood cells (iRBCs) may play a critical role in facilitating the transmission cycle of *Plasmodium* within the human body [25,26]. A proteomic analysis of iRBC-derived EVs revealed distinct sets of virulence-associated proteins depending on the infection stage [27,28]. Recently, Avalos-Padilla et al. proposed a molecular model suggesting that *Plasmodium falciparum* utilizes the endosomal sorting complex required for transport (ESCRT), especially ESCRT-III sub-complex, for delivery to the cytosol of erythrocyte virulence-associated proteins, such as PfBro1, PfVps32 and PfVps60 [29]. These proteins play a crucial role in facilitating the release of EVs from the parasite into the red blood cell cytosol, and subsequently from the cytosol into the extracellular environment.

A comparative analysis of miRNA profiles from EVs derived from infected and non-infected RBCs demonstrated that EVs released by iRBCs are enriched with miRNAs associated with immunomodulatory functions [30]. This study found an upregulation of miR-200a-3p and miR-200b-3p in infected RBC-EVs. Notably, miR-200a-3p is correlated with immune dysfunction of macrophages [31] and CD4^+^ T cells [32], while miR-200b-3p was recently identified as a potent inhibitor of the IFN-γ response induced by viruses [33]. These findings suggest new opportunities to investigate *Plasmodium* immune evasion mechanisms mediated by miRNAs within EVs. As shown, *Plasmodium* species employ EVs as mediators to support the progression of their infectious life cycle and to disrupt host immune mechanisms. EVs originating either from the parasite itself or from iRBCs play a critical role in modulating blood components and other tissues that facilitate infection, including immune cells and the properties of the vascular barrier [34].

### 2.3. Toxoplasma gondii

Approximately one-third of the global human population is infected with *Toxoplasma gondii*, an efficient parasite capable of residing within host cells for the duration of the host’s life [35]. During its asexual cycle, *T. gondii* is capable of infecting any warm-blooded animal, whereas its sexual cycle takes place exclusively in the epithelial cells of the cat gut. In immunocompetent individuals, *T. gondii* infection is usually asymptomatic or causes signs of a mild flu-like illness in the acute stage of infection [36]. However, individuals who are immunodeficient or immunosuppressed and become infected with *T. gondii*—or, in rare instances, experience parasite reactivation following initial clearance by the innate immune system—may develop neurological complications [37]. This occurs as the parasite migrates to the brain, crosses the blood–brain barrier, and subsequently causes damage to the neuronal tissue [38]. An evolutionary factor influences how *T. gondii* manipulates the host immune system. When the parasite remains in the bradyzoite cyst form, it evades detection by host immune cells but does not replicate sufficiently. Conversely, when *T. gondii* shifts to the tachyzoite form, it replicates rapidly but becomes easily detected, potentially causing acute infection that may be fatal in immuno-compromised individuals. Such outcomes stop transmission of *T. gondii* as a species [35].

Although *T. gondii* and toxoplasmosis have been investigated for a long time, the role of EVs in parasite communication with host cells during infection is new to the literature and few studies have so far been carried out. Extensive research conducted by the Pereira-Chiocolla group on the characterization of *T. gondii* EVs has demonstrated that various strains and forms of the parasite have differing cargo profiles. Notably, these EVs consistently contain miRNA molecules that are directly involved in modulating the host immune response, particularly by inducing the presence of inflammatory cytokines such as TNF-α in macrophages [39,40]. The overexpression of miR-21 in EVs released by *T. gondii*-infected microglial cells was considered an oncogenic effector in glioma cells by promoting cancer proliferation and inhibiting the expression of tumor suppressor genes, such as *FoxO1* and *p27* [41]. In addition, contact with *T. gondii* EVs was found to induce cytoskeletal dysfunction in cultured neurons, characterized by a shortening of dendritic extensions and microtubule alterations [42]. These morphological changes were attributed to miR-221-3p, a novel miRNA within these parasite EVs that is known to target and downregulate p27 expression in brain cells [43]. In vitro findings showing that *T. gondii*-derived EVs and associated miRNAs (e.g., miR-221-3p, miR-21) modulate proliferative and tumor-suppressor pathways in glioma and neuronal culture models raise a testable hypothesis regarding a possible link between chronic infection and tumor-associated signaling. However, epidemiological evidence, longitudinal clinical data, and in vivo causal validation in infected human populations are currently insufficient to establish *T. gondii* infection as a risk factor for neuronal cancer, and this association should be regarded as a hypothesis for future investigation rather than an established finding (Figure 1).

*Toxoplasma* cysts in the brain altered the miRNA profile found in neurons after infection, leading these cells to release EVs that contain parasite virulence-associated proteins and miRNAs linked to anti-inflammatory responses [44]. However, in this same study, EVs released by infected neurons were internalized and elicited a proinflammatory response in astrocytes. Another investigation from the Pereira-Chiocolla group showed that EVs derived from *T. gondii* triggered a proinflammatory cytokine profile and an IgG1-like adaptive immune response in infected mice [45]. Similarly, Tawfeek et al. demonstrated that immunization of mice with EVs released by *T. gondii*-infected hepatoblastoma cells conferred protection against subsequent challenge, eliciting enhanced Th1/Th2 immune responses and reducing the brain parasite burden [46]. Furthermore, dendritic cells infected with *T. gondii* were shown to release EVs that polarize macrophages toward an M1 proinflammatory profile in vitro. This EV-mediated immune response is modulated by miR-155-5p, which was found to inhibit intracellular parasite replication through activation of the NF-κB signaling pathway [47]. These findings present new opportunities for further research into the development of a potential vaccine against *T. gondii*, using EVs as a delivery platform.

### 2.4. Leishmania spp.

Leishmaniasis is a term for a group of diseases caused by over 20 species of the parasitic protozoan *Leishmania* spp., affecting an estimated 600,000 to one million people annually in tropical and subtropical regions [48]. The parasite’s life cycle involves both a sandfly vector and a mammalian host. It begins when a female sandfly ingests macrophages containing the non-motile amastigote form during a blood meal from an infected mammal. In the sandfly’s gut, these amastigotes transform into motile promastigotes, which multiply and migrate to the fly’s pharynx. When the infected sandfly bites a human, it injects the promastigotes into the skin. The promastigotes are then phagocytosed by immune cells like macrophages and dendritic cells. This engagement triggers the TLR-9 pathway, stimulating NK cells to produce IFN-γ in an attempt to control the infection [49]. Inside the macrophage, however, the promastigotes transform back into aflagellated amastigotes. Despite being exposed to lethal molecules like nitric oxide, the amastigotes’ high proliferative capacity allows them to survive, multiply, and eventually rupture the host cell to infect other cells [50].

The clinical presentation of leishmaniasis varies by species. Cutaneous leishmaniasis, mainly caused by *L. panamensis* and *L. braziliensis*, is characterized by self-healing skin lesions. Mucocutaneous leishmaniasis, caused by *L. braziliensis*, results from parasite transfer from the skin to mucosal tissue, triggering tissue destruction in the nose, mouth, and pharynx [51]. Visceral leishmaniasis is caused by *L. donovani* and *L. infantum*; it begins with a skin infection, followed by pathogen visceralization that causes chronic fever and hepatosplenomegaly. Currently, there is no approved human vaccine for leishmaniasis—only one for canines to prevent the visceral form. Available drugs are often expensive and have pharmacokinetic limitations [52]. Consequently, different immunotherapies are under development, including cytokine cocktails, monoclonal antibodies, and therapeutic vaccines, a topic recently reviewed by Akbari et al. [53].

Research on the role of EVs in *Leishmania* spp. is a relatively new field. The first description of EVs from this parasite was in 2010 by Silverman et al. [54], and since then, research has focused on the immunomodulatory and drug-resistance properties of these vesicles. In that initial study, a quantitative proteomics analysis showed that EVs from *L. donovani* delivered virulence factors to human macrophages [54]. More recently, EVs from *L. donovani*-infected macrophages were shown to promote cell migration and vascular changes, including angiogenesis, that favor lesion development and the spread of infection [55]. The same research group also revealed that these EVs can induce a gene expression profile in recipient cells that is associated with an anti-inflammatory state [56].

EVs derived from *L. amazonensis* are also a point of interest. This species releases EVs containing GP63, a key virulence glycoprotein, that can stimulate an inflammatory response in macrophages [57]. In this study, using a mouse model of cutaneous leishmaniasis, EV-encapsulated GP63 drove a hyperinflammatory response, worsening the disease outcome [58]. In contrast, other studies have highlighted a protective role for these same EVs. An injection of *L. amazonensis* EVs activated innate-like B-1 cells in mice via the TLR9 pathway [59]. Similarly, a co-injection of EVs and promastigotes promoted immunomodulatory effects in macrophages mediated by TLR4 and TLR2 [60]. Furthermore, EVs from *L. amazonensis*-infected macrophages were able to induce a protective response in naïve macrophages, stimulating the production of IL-12, IL-1β, and TNF-α. This response controlled the infection by reducing the parasite load both in vitro and in vivo [61]. However, the literature currently lacks a detailed characterization of the cargo of EVs derived from infected immune cells, which is essential to elucidate the divergent host responses compared to those triggered by EVs originating directly from *L. amazonensis* (Figure 1).

## 3. Viruses: Extracellular Vesicles in Viral Persistence and Pathology

### 3.1. Human Immunodeficiency Virus

Acquired immunodeficiency syndrome (AIDS) is a severe pathological condition resulting from infection with human immunodeficiency virus type 1 (HIV-1). This enveloped retrovirus is correlated with a global pandemic, with current estimates of more than 40 million living with HIV [62]. The virus employs complex mechanisms to evade and disrupt the host immune system, co-opting immune cells for its replication and dissemination throughout the host. This syndrome is characterized by viral invasion and replication into activated CD4^+^ T lymphocytes, diminishing this cell type population over time, which generates an immunodeficient state in infected individuals [63].

Following transmission, a significant depletion of CD4^+^ T cells occurs within approximately three weeks during the acute infection phase, leading to symptoms such as fever, headache, lymphadenopathy, and skin rash [64]. Subsequently, the viral load in the blood decreases, and the patient’s CD4^+^ T cell population is partially restored as the infection transitions into its chronic stage [65]. This phase is characterized by a gradual loss of CD4^+^ T cells over time, which is induced by immune exhaustion and a persistent inflammatory state in response to the virus [66]. Part of the infected CD4^+^ T cells establish the latent reservoir of HIV, a phenomenon described by Gárcia et al. that involves the transition of these cells to a resting state, resulting in the inhibition of the viral replication to a point that prevents recognition by the host immune system [67,68]. However, the latent reservoir of HIV can rebound the viral infection in cases with antiretroviral therapy (ART) interruption [69].

While considerable progress has been made toward eliminating the HIV reservoir, EVs have recently accumulated interest for their potential role in this effort and other communication processes between cells during each phase of HIV infection. For example, macrophages exposed to EVs released by HIV-positive donors showed an increase in galectin-1 production, which are known for promoting the viral envelope interaction with CD4^+^ T cell receptors and reverse the HIV latency in these cells as well [70]. In addition, dendritic cells were maturated when co-cultured with EVs derived from HIV-infected patients and subsequently reactivated the resting CD4^+^ T lymphocytes. Moreover, HIV-1-infected U937 cells generated EVs capable of reactivating the CD4^+^ T cells latently infected with HIV-1. This process occurred through a mechanism dependent on the production of Nef, a viral protein involved in replication, within the EV-producing U937 cells [71]. These findings indicate that EVs are potential reactivators of the latent HIV reservoir. Although the reactivation of infected lymphocytes might sound dangerous, if the patient is under effective ART, the remote activation of these cells is a key therapeutic strategy, as it would expose the latently infected CD4^+^ T cells that harbor dormant HIV, allowing them to be eliminated by the host immune system in the presence of ART.

The accessory viral protein Nef is a key inducer of the inflammatory state in chronic HIV infection, and EVs derived from infected cells play a fundamental role in delivering this protein to influence other host immune cells [72]. After being internalized by a recipient cell, EV-derived Nef disrupts cholesterol metabolism by reducing levels of the cholesterol transporter ABCA1 [73]. This disruption leads to an accumulation of intracellular cholesterol and a subsequent increase in lipid raft formation [74,75]. The persistent inflammation induced by Nef not only aggravates chronic HIV infection but has also been reported to contribute to neuronal impairments in infected patients [76,77]. This form of EV-mediated communication in HIV pathogenesis is of significant interest, particularly because the presence of Nef in plasma EVs shows potential as a biomarker for chronic inflammation and its associated complications. Furthermore, it was recently observed that the Nef protein is predominantly localized on the outer EV membrane, an observation that led researchers to propose therapeutic strategies to neutralize these Nef-containing vesicles, such as using anti-Nef antibodies [73].

### 3.2. Human Papillomaviruses

Human papillomaviruses (HPVs) are a group of viruses well adapted to their human hosts, using various mechanisms to evade the immune system. Transmission occurs via direct skin or mucosal contact, primarily through sexual activity, making HPV the most common sexually transmitted pathogen worldwide [78]. The initial response to HPV is a classical antigen presenting process that not only contributes to the recruitment and activation of effector cells but also triggers the release of cytokines in order to clear the infection. However, HPV leverages multiple immune evasion strategies, such as altering host cell gene expression and epigenetic profiles [78]. For example, HPV-infected cells can suppress the surface expression of HLA class I molecules, thereby reducing recognition by effector cells and promoting the survival and proliferation of these infected cells [78]. In this way, persistent HPV infection is strongly associated with the development of several types of cancer, most notably cervical and head and neck cancers. The global burden of HPV-associated cancers, which represent about 5% of all malignancies, is most significant in women [79]. Cervical cancer alone ranks as the fourth most common type of cancer and the fourth leading cause of cancer death among women worldwide.

**Figure 2:**
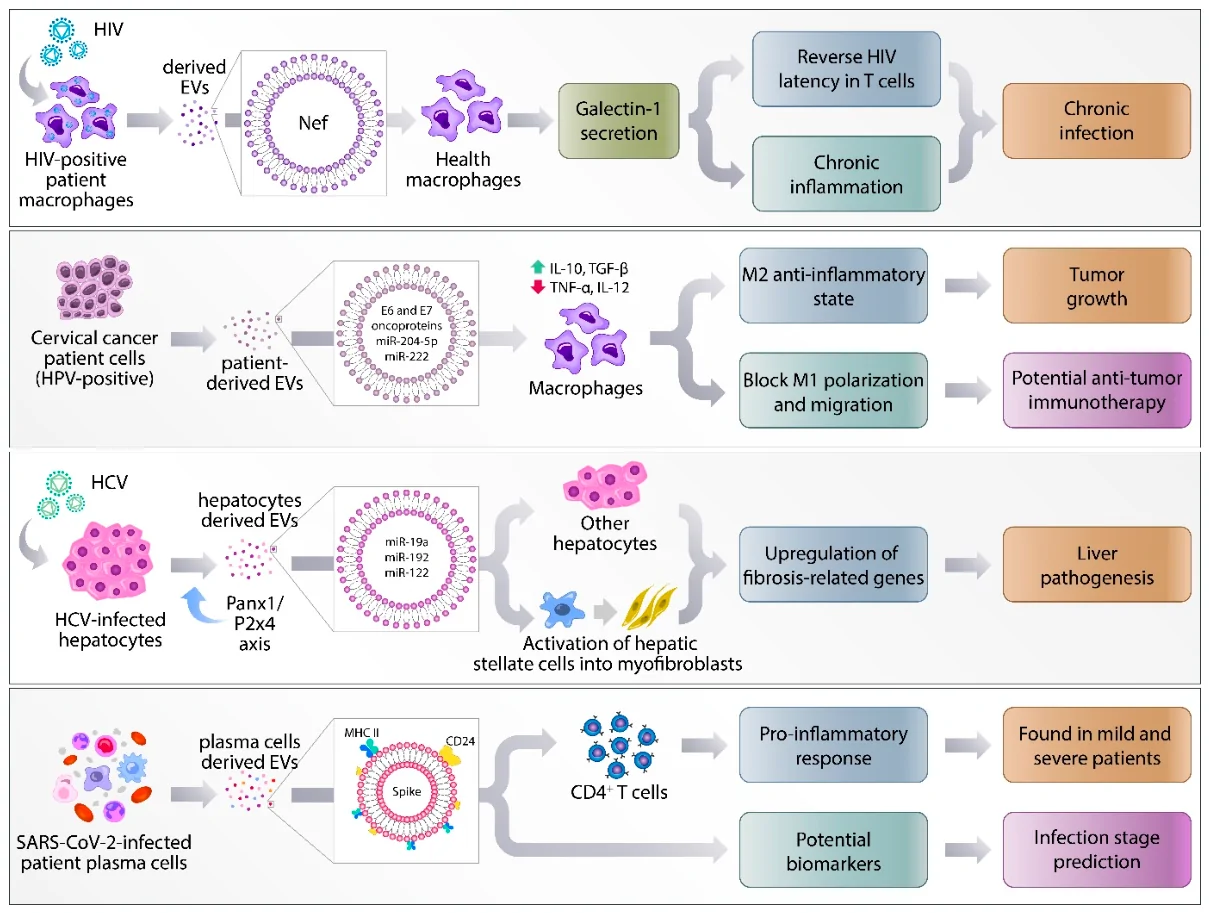
Extracellular vesicle-mediated immune modulation and disease progression in viral infections. Schematic overview of the roles of extracellular vesicles (EVs) in viral pathogenesis, immune regulation, and translational applications. In HIV infection, EVs released from macrophages of HIV-positive individuals carry the viral protein Nef, which is transferred to healthy macrophages, inducing galectin-1 secretion, reversal of HIV latency in CD4^+^ T cells, sustained chronic inflammation, and long-term viral persistence. In HPV-associated cervical cancer, EVs derived from patient tumor cells contain the E6 and E7 oncoproteins and immunomodulatory miRNAs (miR-204-5p and miR-222), which reprogram macrophages toward an M2 anti-inflammatory phenotype, block M1 polarization and migration, and promote tumor growth, while also highlighting EVs as potential platforms for antitumor immunotherapy. During HCV infection, EVs released by infected hepatocytes, regulated by the Panx1/P2X4 axis, deliver miR-19a, miR-192, and miR-122 to neighboring hepatocytes and hepatic stellate cells, driving stellate cell activation, upregulation of fibrosis-related genes, and liver pathogenesis. In SARS-CoV-2 infection, plasma-derived EVs carrying viral Spike protein and immune-related surface markers (including MHC II and CD24) interact with CD4^+^ T cells, inducing pro-inflammatory responses and serving as potential biomarkers associated with disease severity and infection stage prediction. Collectively, this figure illustrates how virus- and host-derived EVs function as critical regulators of immune dysfunction, chronic inflammation, tissue damage, and translational opportunities across diverse viral infections.

The progression from initial infection to invasive cervical cancer, as described by Schiffman et al., involves several critical steps: viral transmission, viral persistence, survival and clone expansion of persistently infected cells leading to carcinogenesis, and finally, invasion [80]. To facilitate immune evasion and tumor progression, HPV-infected and established cancer cells utilize EVs as part of an intercellular communication network (Figure 2). Indeed, recent advances in transcriptomics and proteomics have revealed that EVs contribute to immune disruption by delivering viral transcripts and virulence proteins, such as the E6 oncoprotein, a keystone of HPV-associated cancer development. For instance, Bhat et al. observed that EVs from HeLa cells were enriched with E6 [81]. Following this finding, a more recent study showed that E6-enriched EVs from cervical cancer patients polarized macrophages in vitro towards a M2 anti-inflammatory state, which promotes tumor growth [82]. Moreover, EVs derived from various cervical cancer cells expressing E6 were shown to promote proliferation and angiogenesis in vitro and in vivo by delivering high levels of Wnt7b mRNA to non-cancer cells, activating the β-catenin signaling pathway commonly associated with tumor progression [83]. These findings encapsulate the potential of EVs to act as vectors for viral proteins, thereby promoting HPV infection and associated malignancies.

The HPV oncoprotein E7 is also critical for the development of neoplasia and the promotion of tumor growth. Alongside E6, E7 promotes cell proliferation and inhibits apoptosis. Furthermore, cells infected with HPV strains that are high producers of E6 and E7 release EVs enriched with proangiogenic and immunosuppressive factors capable of modulating cells within the tumor microenvironment [84]. The E6 and E7 oncoproteins alter both the intracellular and the EV-associated miRNA profiles in cervical cancer cells. The intracellular miRNA profile is primarily associated with anti-apoptotic functions, whereas the miRNAs enriched in EVs are involved in modulating tumor-suppressive mechanisms [85]. Interestingly, primary human foreskin keratinocytes transduced to express high levels of HPV16 E7 were found to release EVs carrying miR-222, a miRNA with known pathological roles in many diseases, including cancer [86]. This miRNA is responsible for downregulating the cell cycle inhibitor p27, a homeostatic disruption that has also been observed with EVs derived from *T. gondii*. The convergence of these findings reveals that EVs are alternative mediators capable of crosstalking with and influencing different cell types during tumor development and progression.

On the other hand, over the last decade, E7 has also been investigated for its ability to elicit an immune response. For example, dendritic cells pulsed with HPV16-E7 suppressed macrophage migration and induced M2 polarization. Interestingly, however, EVs derived from these same E7-pulsed dendritic cells promoted the opposite effect, significantly enhancing macrophage migration and inducing polarization towards a proinflammatory M1 state [87]. The mechanism responsible for this differential response needs further investigation; however, it opens up a novel avenue for therapeutic usage of EVs derived from immune cells. For example, Federico’s research group demonstrated that loading HPV oncoproteins into engineered EVs can elicit anticancer immunity against HPV-infected cells in mice. After inoculation, these EVs induced a potent CD8^+^ T lymphocyte response that destroyed target cells and substantially reduced tumor growth [88]. In a subsequent study, this group developed EVs containing anti-HPV16-E7 antibodies. This method not only successfully inhibited intracellular antigens in vitro, but also triggered a bystander effect, in which infected cells that incorporated the antibodies were able to transfer these molecules to nearby cells [89]. These findings are relevant to extend the investigation and efforts to develop a vaccine using EVs as a platform, similar to what has been commented for the usage of *T. gondii* EVs.

### 3.3. Hepatitis C Virus

Hepatitis C virus (HCV) is an enveloped virus that affects approximately 1% of the global population. However, it remains a significant public health problem because no vaccine is available and chronic infection can lead to more threatening diseases, such as hepatocellular carcinoma (HCC) [90]. After transmission, HCV is undetectable for approximately two weeks, with markers of hepatic injury typically appearing after four weeks. Approximately 80% of acutely infected individuals are asymptomatic. When symptoms do occur, they are often non-specific—such as fever, fatigue, vomiting, jaundice, and diarrhea—making a clinical diagnosis based on these signs alone difficult [91]. The acute phase of HCV infection is described in further detail in a recent review by Liu and Kao [91]. The chronic infection stage, which is reached by around 80% of infected individuals, is defined by the persistence of viral load in the blood for more than six months [90].

The antiviral immune response starts when pathogen recognition receptors are activated by viral components inside hepatocytes. This activation triggers a molecular cascade that ends up in the production and secretion of IFNs, which then interact with host immune cells [92]. This IFN-mediated innate response can be escaped by HCV proteins, especially HCV NS3/4 protease complex, which disrupts IFN expression and signaling [93]. In terms of adaptive immune response, patients chronically infected with HCV often display exhausted CD8^+^ T cells and few circulating HCV-specific CD4^+^ T lymphocytes, which can be recovered by treatment [94,95]. Notably, direct-acting antiviral therapy also reshapes the microRNA content of circulating EVs and their immunomodulating properties, suggesting that EV cargo tracks disease and treatment status rather than reflecting a fixed viral signature [96]. As expected, EVs from HCV-infected patients participate in processes that allow the virus to evade host immune surveillance, promote infection, and, in many cases, lead to the development of HCC and fibrosis. For instance, a recent study showed that EVs from the plasma of HCV-infected patients deliver RUNXOR, a long non-coding RNA, to myeloid-derived suppressor cells (MDSCs) [97]. This delivery activated the STAT3 signaling pathway in the MDSCs and increased their production of other suppressive mediators such as arginase 1 and iNOS. In another example, EVs derived from HCV-infected hepatocytes favored the expansion of T follicular regulatory (Tfr) cells, a cell subset commonly found in the liver of patients with chronic HCV infection. These EV-induced Tfr cells then exerted a suppressor function on T follicular helper cells in a TGF-β-dependent manner [98]. These findings highlight an indirect pathway of immune disruption, where EVs transfer information to an intermediate cell type rather than directly influencing the final target cells.

The EV-mediated delivery of miRNAs from HCV-infected cells to other host cells is a fundamental element of intercellular communication (Figure 2). For example, HCV-infected hepatocytes were shown to produce EVs carrying miRNA, such as miR-19a and miR-192, which are associated with the upregulation of fibrosis-related genes in other liver cells in vitro [99,100]. Similarly, a case–control study by Petkevich et al. evaluating the miRNA content of circulating EVs from patients with HCV-related liver diseases found an increase in miR-221-3p compared to healthy volunteers [101]. In addition, EVs isolated from HCV-positive patients were shown to transmit the infection to primary human hepatocyte cultures in a miR-122-dependent manner, a miRNA associated with HCV replication [102,103]. Furthermore, loading these EVs with a miR-122 inhibitor successfully blocked this EV-mediated transmission of HCV. This same miRNA, when packed within EVs from HCV-positive patients, also acts as a key activator of hepatic stellate cells. These cells, which are normally quiescent, become activated and differentiate into myofibroblasts in response to liver injury, leading to fibrosis [104].

Recently, it was suggested that the EV miRNA profile of HCV-infected cells is controlled by the Panx1/P2X4 axis. In this model, Panx1 is responsible for EV formation and release, while P2X4 mediates calcium influx, promoting an inflammatory state in hepatocytes and inducing the activation of hepatic stellate cells into myofibroblasts [105,106]. Typically, this cellular transformation occurs as a reaction to liver damage, prompting myofibroblasts to produce various types of collagen. If apoptosis does not remove these myofibroblasts, they can contribute to liver fibrosis and promote inflammation by building up pro-inflammatory cytokines in the affected area [107]. To support this transformation of hepatic stellate cells modulated by EVs, the same study demonstrated that inhibiting Panx1 and P2X4 prevented the accumulation of high miRNA levels, particularly miR-122, in EVs derived from HCV-infected hepatocytes [104].

Multiple analyses have identified miR-122 as a factor with high diagnostic value for predicting HCV therapy efficacy, as its levels are closely correlated with treatment response and patient outcome [108]. In addition, using logistic regression analysis, the expression of miR-122 in circulating EVs from individuals with different liver fibrosis stages was found to be directly correlated with disease severity [104]. Furthermore, a broader correlation between the levels of circulating miR-122 and liver injury in general has been observed in patients with various liver complications [109]. Many researchers suggest that the miRNA signature found in HCV-infected individuals can be used as a non-invasive biomarker for diagnosing HCV and monitoring other potential liver complications [104,109]. While miRNAs are commonly proposed for this role, other types of RNAs found in patient-derived EVs, including mRNAs and some long non-coding RNAs, might also serve as biomarkers for HCV infection and liver injury [110,111].

### 3.4. SARS-CoV-2

The outbreak of the COVID-19 pandemic underscored the significant threat that airborne and respiratory pathogens pose to global health. Contrary to the common perception that these viruses cause only mild, flu-like symptoms and signs, persistent infections can lead to more complicated diseases, including lung injuries and acute respiratory syndromes. The immune response to respiratory virus infection is typically effective. However, the alveolar inflammation required for viral clearance can become damaging if it is prolonged. This harmful process involves the activation of many immune cells—such as macrophages, neutrophils, and CD4^+^ T helper lymphocytes—coupled with a sustained release of high-level proinflammatory cytokines and virulence mediators in the lungs [112]. The innate response to a respiratory virus infection is IFN-mediated, similar to that of an HCV infection. This process involves a cascade of molecular events that leads the immune response after infected cells release IFNs into the extracellular space. The overall pathogenesis of a respiratory virus infection is also influenced by other factors, including the inflammatory response itself, the viral load, the host’s age and immune repertoire, and the virus’s virulence. The innate and adaptive immune responses during respiratory virus infection are reviewed in detail by Stambas et al. [113].

The release of EVs from infected airway epithelial and immune cells plays a role in regulating pulmonary immune surveillance during the infection. As one of the most studied recent viruses, SARS-CoV-2 is a respiratory pathogen that caused over 7 million deaths in a short period [114]. In the context of COVID-19, the cargo and surface components of EVs differ depending on the infection’s severity. EVs from cells infected with SARS-CoV-2 reportedly act as key mediators between the infection and the immune response. For instance, while EVs from the plasma of mildly infected individuals carry MHC class II molecules on their surface to promote a proper CD4^+^ T cell response, EVs from patients with severe COVID-19 displayed fewer of these molecules and a higher content of inflammatory modulators [115]. Moreover, studies have investigated the correlation between specific EV-derived biomarkers and COVID-19 severity. For example, CD24 on EVs isolated from the sera of infected patients has been proposed as a biomarker [116]. In this study, CD24^+^ EVs were present in patients with mild symptoms but absent in those with severe disease. This result suggests that individuals whose EVs lack CD24 early in an infection may be more prone to developing severe symptoms. On the other hand, EVs derived from the blood of patients with severe COVID-19 expressed high levels of CD41A, CD31, CD142, and CD162 [117], which are markers related to inflammatory responses. In another study, EVs from the plasma of COVID-19 patients showed an abundance of the platelet marker CD41 [118]. Since platelet-derived EVs are common in severe infections and are linked to the development of thrombosis, this finding suggests their involvement in the thrombotic complications of COVID-19 [119]. Combining early and late EV biomarkers for COVID-19 seems to be a powerful way to determine a patient’s infection stage and predict the potential for disease progression to more complicated symptoms.

EVs released by SARS-CoV-2-infected cells can also promote viral infection by delivering viral proteins to different tissues and organs (Figure 2). The Spike protein is a key component of SARS-CoV-2, responsible for viral binding and entry into host cells [120]. This protein has been found in EVs released from the cells of infected patients, and this form of intercellular communication may allow the virus to evade immune surveillance [121,122,123]. For example, these Spike-containing vesicles have been reported to act as decoys that neutralize antibodies, thereby reducing the host’s immune protection against the virus [121,122]. Due to the high affinity between Spike and ACE2 receptors, which are expressed in multiple tissues [124], the presence of Spike in EVs has been suggested as a non-invasive way to monitor and characterize affected organs [123,125]. The Spike protein encapsulated within EVs was recently reported in the plasma of patients with post-acute sequelae of COVID-19 at levels similar to those found during the acute phase of infection, a finding which may explain the prolongation of symptoms [126]. This result sheds light on the persistence of EVs with viral components in the body long after pathogen clearance, which may contribute to the development of sequelae in infected individuals.

Furthermore, it is worth noting that patients with post-acute sequelae of COVID-19 have a higher risk of developing cardiovascular complications [127]. Consistent with this, persistent circulating EVs containing Spike have been reported to exaggerate heart dysfunction in a mouse model of pre-existing heart failure [128]. Another study revealed that EVs released by SARS-CoV-2-infected cells can interact with hepatocellular carcinoma cells in vitro, leading to tumor progression and metastasis [129]. Taken together, these findings underscore the need for new therapeutic strategies, including next-generation vaccines, designed to eliminate circulating EVs that carry viral proteins and contribute to long-term sequelae.

The use of EVs as a therapy for severe COVID-19 has been proposed, with a particular focus on those derived from mesenchymal stromal/stem cells (MSCs). MSCs themselves have been trialed as a treatment for various inflammatory diseases [130], such as lung fibrosis [131], and their secreted EVs are known to have anti-inflammatory properties [132]. Supporting this approach, a double-blind, randomized, controlled clinical trial using MSC-derived EVs showed potential in decreasing the mortality rate of hospitalized patients with severe COVID-19 [133]. Another randomized clinical trial reported similar outcomes, demonstrating that treatment with bone marrow MSC-derived EVs significantly reduced risk in all tested individuals compared to a placebo group [134]. Moreover, this cell-free EV therapy has shown promise for treating not only COVID-19 but also influenza virus infections. Additionally, adipose-tissue MSCs release EVs that are capable of preventing lung injury induced by SARS-CoV-2 and H1N1 influenza virus in vitro and in animal models [135]. For instance, in another in vitro study, MSC-EVs rich in miR-146a-5p were administered to influenza-infected lung cells and induced the downregulation of inflammatory cytokine expression in these cells by modulating the NF-κB signaling pathway [136].

Several ongoing clinical trials are exploring MSC EV-based treatments and vaccine development; this topic is reviewed in greater detail by Mizenko et al. [137]. In summary, these findings suggest that just as EVs can play an important role in promoting infection, they can also be used as a powerful and safe tool to design new therapies against current airborne pathogens and future emerging viruses.

## 4. Bacteria: Dual Roles of Extracellular Vesicles in Host Defense, Pathogenesis, and Immune Remodeling

### 4.1. Mycobacterium tuberculosis

As a leading cause of death from an infectious pathogen, *Mycobacterium tuberculosis* was responsible for over 1.23 million deaths worldwide in 2024 [138]. The risk of tuberculosis begins when the airborne bacteria are inhaled and engulfed by alveolar macrophages in the lungs [139]. During the innate immune response, infected macrophages trigger IFN responses to recruit other immune cells, forming a granuloma. This structure, a “wall” surrounding the infected cells, is initially a host-protective barrier. The typical granuloma is composed of infected macrophages, neutrophils, eosinophils covered by T and B cells that express pro-inflammatory and anti-inflammatory cytokines [140]. However, the bacterium exploits the granuloma to promote the infection, and over time, the immune system fails to completely eradicate the pathogen [141]. Surviving infected cells can then move to the lymph nodes, proliferate, and access the bloodstream, leading to dissemination to other organs [142]. *M. tuberculosis* can establish a persistent infection that remains asymptomatic in the host, a phase known as latent tuberculosis, which affects over two billion people globally [138,143], a topic well reviewed in detail by Boom et al. [144].

**Figure 3:**
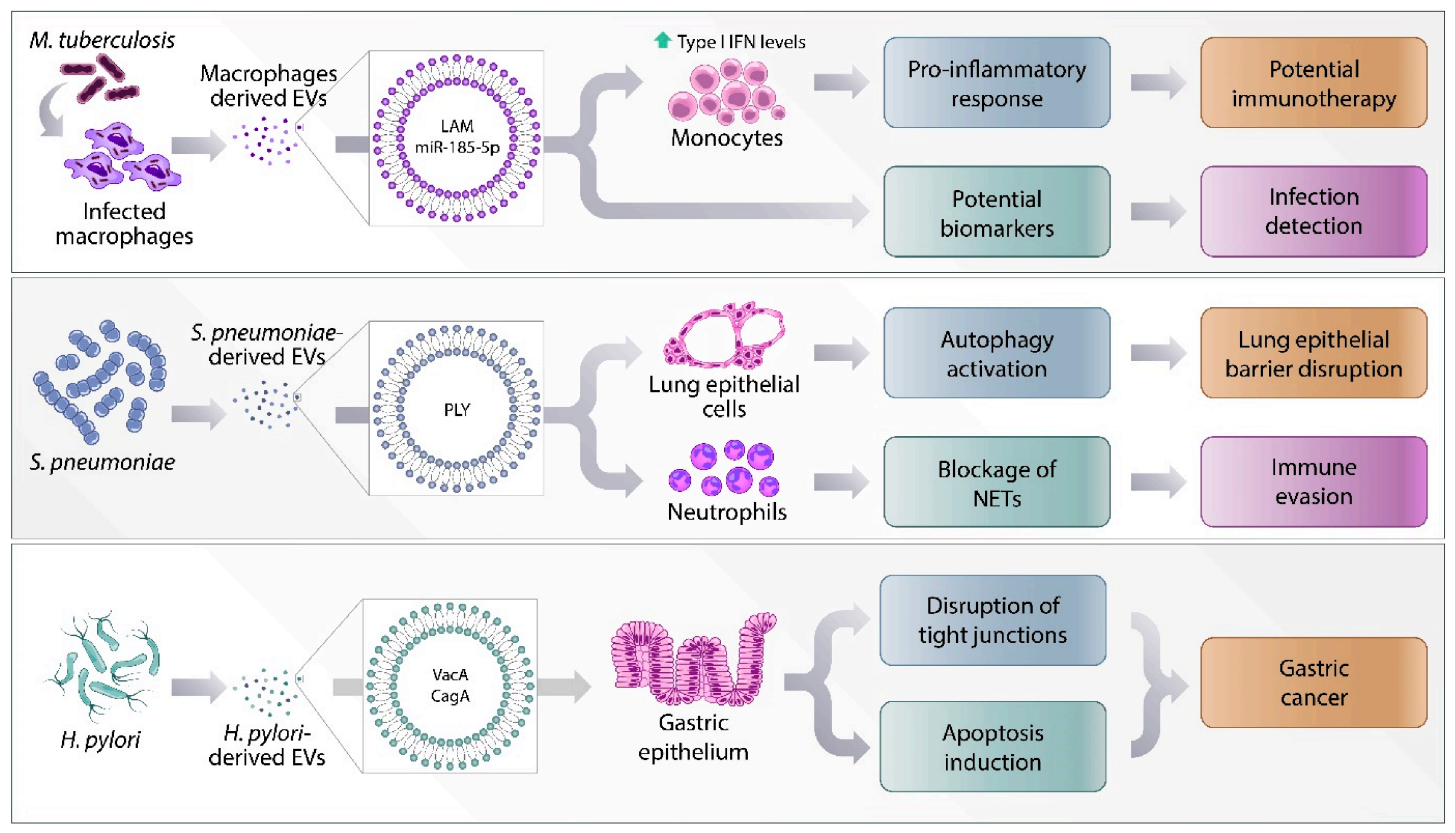
Extracellular vesicle-mediated immune modulation and pathogenesis in bacterial infections. Schematic representation of the diverse roles of extracellular vesicles (EVs) in bacterial infections, highlighting their contribution to immune activation, immune evasion, tissue damage, and translational applications. In *Mycobacterium tuberculosis* infection, EVs released from infected macrophages contain mycobacterial components such as lipoarabinomannan (LAM) and host-derived miR-185-5p, which stimulate monocytes, elevate type I interferon (IFN) levels, and promote pro-inflammatory responses. These EVs also represent promising candidates for non-invasive biomarkers and host-directed immunotherapeutic strategies for tuberculosis detection and treatment. In *Streptococcus pneumoniae* infection, bacterial-derived EVs enriched with pneumolysin (PLY) interact with lung epithelial cells and neutrophils, triggering autophagy activation, disrupting the lung epithelial barrier, blocking neutrophil extracellular trap (NET) formation, and facilitating immune evasion. In *Helicobacter pylori* infection, pathogen-derived EVs deliver the virulence factors VacA and CagA to gastric epithelial cells, leading to disruption of tight junctions, induction of apoptosis, and progressive gastric pathology that contributes to gastric carcinogenesis. Collectively, this figure underscores the multifaceted and context-dependent functions of bacterial EVs in shaping host immune responses, tissue integrity, and disease outcomes, while emphasizing their potential as diagnostic and therapeutic targets.

Almost twenty years ago, a study using *M. tuberculosis* as a model reported that intercellular communication between infected and uninfected cells contributes to pathogen transmission and disease development [145]. The authors suggested that EVs from *M. tuberculosis*-infected macrophages delivered mycobacterial components, such as lipoarabinomannan (LAM) to mice models and to uninfected human monocytes in vitro, stimulating a pro-inflammatory response (Figure 3). Recently, EVs from *M. tuberculosis*-infected macrophages were shown to induce a type I IFN response in uninfected macrophages [146]. This response, triggered by pathogen-associated molecular patterns (PAMPs), suggests the EVs were loaded with immunostimulatory bacterial components. The same study also proposed an immunotherapeutic strategy combining these pro-inflammatory EVs with antibiotics. This approach synergistically decreased the bacterial load in infected macrophages in vitro and reduced the bacterial burden in the lungs of *M. tuberculosis*-infected mice.

The protein profile of EVs from infected macrophages differs significantly from that of EVs from uninfected control cells. For instance, a characterization study by Diaz et al. revealed a novel intercellular communication pathway involving these vesicles. They found that EVs from infected macrophages were loaded with antimicrobial molecules, such as heat-shock proteins, suggesting they function as part of a host defense mechanism [147]. Furthermore, EVs from *M. tuberculosis*-infected MSCs can trigger pro-inflammatory responses in macrophages via TLR2 and TLR4. This effect was observed both in vitro and in vivo, where the response led to increased neutrophil infiltration in the lungs [148]. In a related study, EVs from *M. tuberculosis*-infected neutrophils were also shown to promote pro-inflammatory cytokine release from human macrophages, a response that ultimately reduced intracellular bacterial viability [149]. Additionally, EVs from infected neutrophils reportedly contain mycobacterial antigens that facilitate dendritic cell maturation, thereby leading to the activation of a Th1 lymphocyte response by producing high levels of IFN-γ [150]. Similarly, EVs isolated from the serum of infected mice have been shown to activate endothelial cells via the same IFN pathway. This activation led to the upregulation of immune-related genes, including TLR2, in the endothelial cells [151]. These findings offer a different perspective on the role of EVs in *M. tuberculosis* infection. It seems that infected cells can release EVs that prioritize host defense by delivering antigens for presentation to other immune cells or by transmitting molecular signals to trigger an adaptive immune response.

The presence of bacterial components in EVs from *M. tuberculosis*-infected cells makes them promising candidates for non-invasive tuberculosis biomarkers, potentially facilitating disease diagnosis. For instance, one cohort study found high levels of the mycobacterial component LAM in circulating EVs from tuberculosis-positive children, which could be detected with simple methods [152]. Another blood-based test proposed the use of a specific miRNA, miR-185-5p, as a potential biomarker, as it was found in EVs from both the plasma of patients with tuberculosis and from infected macrophages in vitro [153]. Furthermore, proteomic studies have revealed altered protein profiles in EVs from tuberculosis patients. A case–control study, for example, identified differentially expressed proteins in EVs from tuberculosis-positive individuals compared to uninfected controls. This notably included a decreased expression of proteins related to lipid metabolism, such as APOA1 [154]. Low levels of this protein may lead to cholesterol accumulation in alveolar macrophages, thereby reducing their phagocytic capacity [155]. These findings highlight lipid metabolism pathways as a source of novel biomarkers for pulmonary tuberculosis.

### 4.2. Streptococcus pneumoniae

*Streptococcus pneumoniae* represents a major health problem, as this encapsulated gram-positive bacterium can cause pneumonia and meningitis. The polysaccharide capsule of *S. pneumoniae*, first described by Pasteur in 1880, is crucial for virulence and the spread of infection within the host, allowing the pathogen to evade host immune defenses [156]. Transmission of this airborne bacterium occurs primarily between humans, typically through respiratory droplets. The infection begins with the colonization of the nasopharynx by *S. pneumoniae*. From this initial site, the bacteria can translocate to other parts of the body, including the lungs, bloodstream, and central nervous system [157]. Pneumonia caused by *S. pneumoniae*, known as pneumococcal pneumonia, is characterized by strong lung inflammation. This inflammation results from a strong pro-inflammatory response to the infection, which includes the recruitment of immune cells to the lungs [158]. While pneumococcal disease is preventable with vaccines and treatable with antimicrobial therapy, pneumonia in general remains a significant global health issue, causing an estimated 730,000 deaths in children annually [159].

Research into the role of EVs in *S. pneumoniae* infection is relatively new, with only about a decade of studies on the topic and few entries. However, published results indicate that *S. pneumoniae* can modulate host cells either via its own vesicles or by hijacking the host’s vesicle formation machinery to transfer virulence factors to other cells. Recently, EVs from different *S. pneumoniae* strains were found carrying bacterial DNA on their outer surface. These EVs can interact with other bacterial cells, serving as a source of DNA for recombination and thereby increasing resistance within the bacterial population [160]. Besides carrying DNA, *S. pneumoniae* EVs can also carry virulence proteins. For example, a study revealed that these EVs are enriched with pneumolysin (PLY), a pore-forming toxin, that was delivered to lung epithelial cells. This delivery activated dendritic cells and induced a pro-inflammatory response [161]. In the same study, the EVs also acted as decoys by binding to complement proteins, thereby disrupting phagocytosis-mediated killing by macrophages. EVs from *S. pneumoniae* have been described as crucial for interacting with host cells, activating the autophagy pathway in the alveolar epithelial cells of mice [162]. The lung epithelial barrier was disrupted by kinases within these EVs. These kinases are responsible for phosphorylating autophagy-initiation proteins, which in turn triggers the degradation of occludin, an essential structural component of the epithelium.

*S. pneumoniae*-infected cells can release EVs that modulate other host cells to sustain the infection and evade immune defenses (Figure 3). For example, the virulence protein PLY modulated EV production in infected neutrophils to cause platelet activation and aggregation, a phenomenon that is predictive of a poor outcome for patients with pneumonia [163]. As another immune evasion strategy, EVs from *S. pneumoniae* delivered a DNAse to neutrophils which is capable of compromising the formation of neutrophil extracellular traps (NETs), a classical mechanism of innate immune response against bacterial infection, by using the DNA to detain and kill the pathogen [164]. In contrast, other studies have revealed that EVs can also induce protective adaptive immune responses. For example, EVs from the dendritic cells of infected mice were shown to carry capsular polysaccharide antigens, which stimulated antibody production by B cells [165]. The study demonstrated that vaccination with these EVs induced specific immunoglobulin production and protected the animals against a lethal challenge with a virulent *S. pneumoniae* strain. Another study proposed the usage of EVs as a cell-free vaccination against *S. pneumoniae*, this time using the EVs released by the pathogen, which were responsible for eliciting a potential immunostimulatory effect by increasing the dendritic cell production of TNF-α [166].

### 4.3. Helicobacter pylori

Almost half of the world’s population has *Helicobacter pylori* colonizing their gastric mucosa [167]. Transmission of this spiral-shaped, gram-negative bacterium typically occurs via oral–oral or fecal–oral routes during childhood, and the infection can persist for a lifetime [168]. The persistence of *H. pylori* leads to chronic gastritis, a known high-risk factor for gastric cancer. It can also lead to the development of other diseases, such as gastric mucosa-associated lymphoid tissue (MALT) lymphomas and gastric ulcers [169,170,171]. Despite its prevalence, most infected individuals remain asymptomatic, as *H. pylori* successfully colonizes the stomach and modulates the immune system to maintain a chronic inflammatory state [168]. The innate immune response to *H. pylori* involves multiple pathways, including the activation of NF-κB and the recruitment of neutrophils, macrophages, and dendritic cells to the infected mucosa. However, *H. pylori* has evolved sophisticated evasion strategies. Interestingly, it possesses a structurally distinct lipopolysaccharide (LPS) that is poorly recognized as a PAMP, allowing the bacterium to evade innate immune detection [168]. The adaptive immune response is also incapable of clearing the infection; even when antibodies are produced against *H. pylori*, they have little effect on the bacterial population [172].

A classic article by Fiocca et al. first proposed that *H. pylori* releases its virulence factors via EVs, showing that vacuolating toxin A (VacA) was taken up by the gastric epithelium [173]. Since then, both VacA and cytotoxin-associated gene A (CagA)—a pathogenic factor strongly linked to gastric cancer—have been identified in EVs from *H. pylori*. These vesicles serve as a key mechanism for delivering these virulence factors to host cells (Figure 3). For example, EVs rich in CagA have been found to disrupt tight junctions in epithelial cells, a phenomenon associated with cancer progression [174]. The effectiveness of this EV-delivered CagA depends on its ability to evade autolysosomal degradation within the host cell [175]. Similarly, VacA delivered by EVs plays multiple roles. It can trigger apoptosis in both gastric epithelial cells and T cells [176,177] and can also contribute to carcinogenicity by causing glutathione depletion and genomic instability in vitro [178]. Taken together, these findings reflect the ability of *H. pylori* to use EVs to deliver its virulence factors to host cells, thereby orchestrating multiple pathways that disrupt the gastric mucosa.

The systemic effects of *H. pylori* infection are mediated by EVs originating not only from the bacterium itself, but also from infected host cells. These cells can package and spread pathogenic factors using the host cells’ vesicle machinery. For example, EVs carrying CagA from *H. pylori*-infected gastric epithelial cells were found to suppress the transcription of cholesterol efflux transporters in macrophages. This mechanism, similar to the one described for HIV, led to lipid accumulation within the macrophages, thereby promoting atherosclerosis [179]. CagA-dependent morphological changes in epithelial cells induced by *H. pylori*-infected cells can trigger a phenotypic change to a more elongated shape, resembling a mesenchymal form [180]. Furthermore, studies of *H. pylori*-infected bone marrow mesenchymal stem cells (MSCs) revealed that they release EVs capable of transferring biological information, such as thrombospondin-2—a glycoprotein associated with angiogenesis, also known as a prognostic marker of colorectal cancer [181]. EVs from these infected MSCs were shown to enhance the proliferation, migration, and invasion of gastric cancer cells in vitro, as well as promote carcinogenesis and metastasis in mouse xenografts [182]. EVs can also promote cancer progression through an indirect mechanism involving macrophages. In one study, *H. pylori*-infected gastric cells transferred mesenchymal–epithelial transition (MET) factor to macrophages via EVs. Upon exposure to these EVs, macrophages shifted to an infiltrating phenotype in vitro and facilitated gastric tumor growth in vivo—an infiltration pattern that is associated with a poor prognosis in gastric cancer patients [183].

## 5. Convergent and Divergent EV-Mediated Mechanisms Across Pathogen Kingdoms: Toward a Unifying Framework

Beyond the pathogen-by-pathogen findings summarized above, direct comparison across the protozoan, viral, and bacterial sections reveals recurring principles as well as fundamental points of divergence that, together, constitute the unifying framework this review set out to establish.

### 5.1. Convergent Strategies

Three mechanistic themes recur across all three pathogen kingdoms despite their profound biological differences. First, EV cargo consistently exhibits a dual, context-dependent capacity to either promote or restrain infection. This duality is evident in protozoan infection, where *L. amazonensis* EVs can be either hyperinflammatory or protective depending on the experimental context [58,61]; in mycobacterial infection, where EVs from *M. tuberculosis*-infected cells can carry either immunostimulatory PAMPs [145,146] or antimicrobial, host-protective cargo [147,148,149,150,151]; and in HIV infection, where EVs can both reactivate latent provirus [70,71] and, in other contexts, restrict viral replication. Second, small non-coding RNAs delivered via EVs emerge repeatedly as a shared mechanistic currency for reprogramming recipient cells, from *T. gondii* miR-221-3p silencing p27 in neurons [44] to HCV-associated lncRNA RUNXOR activating STAT3 signaling in myeloid-derived suppressor cells [97] to *M. tuberculosis*-associated miR-185-5p in circulating EVs [153]. Third, across all three kingdoms, EV cargo, whether pathogen-derived antigens, virulence factors, or host response signatures, consistently shows translational promise as a non-invasive biomarker, a theme that recurs independently in the protozoan, viral, and bacterial literature reviewed here.

### 5.2. Divergent Strategies

Alongside these convergences, the three pathogen kingdoms rely on topologically and mechanistically distinct strategies that a kingdom-siloed reading of the literature obscures. Protozoan EVs primarily act locally, modulating the cytokine milieu and cytoskeletal architecture of directly exposed host cells [39,40,42]; their cargo is dominated by parasite-specific glycoproteins (e.g., GP63 [57,58]) and microRNAs, with comparatively little evidence, to date, of systemic redistribution. Viral EVs, in contrast, exploit the host ESCRT and endosomal machinery to disseminate viral proteins and genomic material well beyond the site of infection, acting as decoys for neutralizing antibodies [121,122] and as vehicles for long-range reactivation of latent reservoirs [70,71], a strategy without a clear counterpart among the protozoan or bacterial pathogens discussed here. Bacterial EVs are mechanistically distinct at their origin: whether generated as classical outer membrane vesicles or as host-derived vesicles enriched in bacterial virulence factors such as LAM [145], PLY [163,164], CagA, and VacA [173,174,175,176,177,178], they characteristically deliver microbial pathogen-associated molecular patterns and toxins directly recognized by host pattern-recognition receptors, a route of immune engagement with no direct equivalent among the eukaryotic pathogen EVs discussed above.

### 5.3. Unresolved Questions and Methodological Gaps

Reading across the three pathogen groups also exposes gaps that individual, pathogen-restricted reviews rarely make explicit. The molecular determinants of cargo sorting into pathogen- or host-derived EVs remain poorly defined for the large majority of the molecules discussed in this review, and it is generally unknown whether the same sorting machinery operates similarly across protozoa, viruses, and bacteria. Most mechanistic findings, in every pathogen group, derive from in vitro systems using isolated cell lines or short-term murine models; the extent to which these findings translate to chronic, natural human infection, particularly for pathogens capable of decades-long latency such as *M. tuberculosis* and HIV, remains largely unverified. Finally, EV isolation and characterization methods vary considerably across the studies compiled here (density-gradient ultracentrifugation, size-exclusion chromatography, or commercial precipitation-based kits), a methodological heterogeneity that complicates direct comparison of cargo composition and yield between studies and pathogen groups, and that future work in this field should address through greater adherence to community reporting guidelines such as MISEV.

The EV-based vaccine strategies discussed throughout this review fall into at least four categories with distinct safety and regulatory considerations: natural pathogen-derived vaccines (e.g., *Leishmania* or *Trypanosoma* exosome-like vesicles, bacterial outer-membrane vesicles), which carry a risk of unintended virulence-factor transfer; infected-host-cell EV vaccines; immune-cell EV vaccines, such as dendritic-cell-derived EVs loaded with antigen ex vivo; and engineered EV delivery systems, frequently based on MSC or immune-cell EVs modified to display antigens or adjuvants, for which batch-to-batch variability, scalability, and off-target biodistribution represent the main manufacturing and regulatory challenges. These categories should not be regarded as interchangeable despite their shared vesicular nature.

Taken together, this comparative synthesis, rather than a further pathogen-by-pathogen summary, constitutes the principal conceptual contribution of this review: it identifies which EV-mediated strategies are genuinely conserved across infectious agents from different kingdoms, which are kingdom-specific adaptations, and which questions cannot be answered from the pathogen-siloed literature alone.

## 6. Future Perspectives and Translational Challenges

The findings compiled throughout this review indicate that EVs are neither passive infection byproducts nor a pathogen-specific curiosity, but a conserved communication layer with real diagnostic and therapeutic potential. Realizing that potential, however, requires overcoming several experimental, biological, and regulatory obstacles that remain largely unresolved.

A first challenge is standardization. EV isolation protocols differ substantially in yield, purity, and subpopulation bias across the ultracentrifugation-, precipitation-, and chromatography-based methods used in the studies discussed above, complicating cross-study comparison of cargo composition and limiting reproducibility, an issue increasingly recognized by the field and addressed by consensus guidelines such as MISEV. A second challenge concerns specificity: many pathogen-associated EV cargoes described here (e.g., LAM-positive EVs in tuberculosis [152], miR-185-5p [153], or circulating Spike-containing EVs in COVID-19 [123,125,126]) still require validation in large, prospective, and geographically diverse patient cohorts before their diagnostic performance can be established relative to existing gold-standard assays. Third, the precise molecular machinery governing selective cargo loading into EVs, which determines why a given microRNA, protein, or virulence factor is enriched in pathogen- or host-derived EVs rather than randomly incorporated, remains incompletely characterized for essentially every pathogen discussed in this review, and resolving this question is a prerequisite for the rational engineering of EV-based vaccines and therapeutics.

On the therapeutic side, EV-based interventions face additional hurdles beyond those of conventional biologics. Scalable, GMP-compliant manufacturing of clinical-grade EVs, whether derived from mesenchymal stromal cells [130,131,132,133] or engineered to display specific antigens, remains technically demanding and costly. Regulatory frameworks for EV-based products are still evolving, and no consensus yet exists on potency assays, dosing, or long-term safety monitoring specific to EV therapeutics. Furthermore, because EV cargo composition depends on the physiological and inflammatory state of the producer cell, engineered or cell-derived EV therapeutics may behave unpredictably across the heterogeneous inflammatory environments seen in different infections and patient populations, an issue that will require careful pharmacodynamic characterization before broader clinical adoption.

Addressing these challenges will require coordinated efforts across microbiology, extracellular vesicle biology, bioengineering, and regulatory science. We anticipate that the next generation of studies in this field will increasingly integrate multi-omic characterization of EV cargo with functional validation in physiologically relevant, ideally human-derived, infection models, moving the field from mechanistic description toward validated clinical application.

## 7. Conclusions

EVs have emerged as central and versatile mediators of host–pathogen communication across a wide spectrum of infectious diseases. As highlighted in this review, protozoan parasites, viruses, and bacteria exploit EVs either by releasing pathogen-derived vesicles or by hijacking host EV biogenesis pathways to modulate immune responses, promote immune evasion, disseminate virulence factors, and sustain infection. Despite the remarkable biological diversity of these pathogens, common EV-driven strategies recur, underscoring the existence of conserved mechanisms through which infections reshape host cellular networks.

Beyond their role in pathogenesis, EVs represent a critical interface between infection and host immunity, capable of driving divergent outcomes ranging from protective immune activation to chronic inflammation, tissue damage, and oncogenic transformation. The context-dependent nature of EV cargo composition, shaped by pathogen species, life cycle stage, infection severity, and host cell type, is emerging as a key determinant of disease progression and clinical outcome. However, significant knowledge gaps remain, particularly regarding the precise molecular sorting mechanisms governing EV cargo loading and the functional heterogeneity of EV subpopulations in vivo.

Importantly, the translational potential of EVs is becoming increasingly evident. Circulating EV-associated molecules offer promising avenues for the development of non-invasive biomarkers for diagnosis, prognosis, and therapeutic monitoring. In parallel, advances in EV engineering and cell-free therapies position these vesicles as innovative platforms for vaccine development and immunomodulatory interventions against infectious diseases. It is important to note that, for most pathogens discussed here, the relative contribution of EV-mediated communication compared with other mechanisms of host–pathogen interaction, including soluble cytokines, direct cell–cell contact, free pathogen products, virions, and tissue-level processes, has not been formally established.

In summary, EVs redefine host–pathogen interactions as a dynamic, vesicle-mediated communication system, positioning EVs not merely as byproducts of infection but as strategic molecular hubs whose targeting and exploitation may fundamentally reshape the future of infectious disease diagnosis, prevention, and therapy.

## Data Availability

No new data were created or analyzed in this study. Data sharing is not applicable to this article.
